# Construction of reporter gene assays using *CWP* and *PDR* mutant yeasts for enhanced detection of various sex steroids

**DOI:** 10.1186/s41021-020-00159-x

**Published:** 2020-05-27

**Authors:** Sayoko Ito-Harashima, Mami Matano, Kana Onishi, Tomofumi Nomura, Saki Nakajima, Shingo Ebata, Kazuhiro Shiizaki, Masanobu Kawanishi, Takashi Yagi

**Affiliations:** 1 Department of Biological Sciences, Graduate School of Science, Osaka Prefecture University, 1-2 Gakuen-cho, Naka-ku, Sakai, Osaka, 599-8570 Japan; 2 Present address: Department of Applied Biosciences, Graduate School of Life Sciences, Toyo University, 1-1-1 Izumino, Itakura-machi, Ora-gun, Gunma 374-0193 Japan

**Keywords:** Estrogen receptor, Androgen receptor, Progesterone receptor, Steroid hormone, *Saccharomyces cerevisiae*, Reporter gene assay, Agonist, Antagonist, Inverse agonist

## Abstract

**Background:**

Sex steroid hormone receptors are classified into three classes of receptors: estrogen receptors (ER) α and β, androgen receptor (AR), and progesterone receptor (PR). They belong to the nuclear receptor superfamily and activate their downstream genes in a ligand-dependent manner. Since sex steroid hormones are involved in a wide variety of physiological processes and cancer development, synthetic chemical substances that exhibit sex steroid hormone activities have been applied as pharmaceuticals and consumed in large amounts worldwide. They are potentially hazardous contaminants as endocrine disruptors in the environment because they may induce inappropriate gene expression mediated by sex steroid hormone receptors in vivo.

**Results:**

To develop simple reporter gene assays with enhanced sensitivity for the detection of sex steroid hormones, we newly established mutant yeast strains lacking the *CWP* and *PDR* genes encoding cell wall mannoproteins and plasma membrane drug efflux pumps, respectively, and expressing human ERα, ERβ, AR, and PR. Reporter gene assays with mutant yeast strains responded to endogenous and synthetic ligands more strongly than those with wild-type strains. Sex steroid hormone activities in some pharmaceutical oral tablets and human urine were also detectable in these yeast assays.

**Conclusions:**

Yeast reporter gene assay systems for all six steroid hormone receptors, including previously established glucocorticoid receptor (GR) and mineralocorticoid receptor (MR) assay yeasts, are now available. Environmental endocrine disrupters with steroid hormone activity will be qualitatively detectable by simple and easy procedures. The yeast-based reporter gene assay will be valuable as a primary screening tool to detect and evaluate steroid hormone activities in various test samples. Our assay system will strongly support the detection of agonists, antagonists, and inverse agonists of steroid hormone receptors in the field of novel drug discovery and assessments of environmental pollutants.

## Introduction

The sex steroid hormones, estrogens, androgens, and progesterone, play essential roles in sexual differentiation, reproduction, and many other physiological processes by regulating gene expression in vertebrates [1–4]. The receptors of these steroids, estrogen receptors α and β (ERα and ERβ), androgen receptor (AR), and progesterone receptor (PR), belong to the nuclear receptor (NR) superfamily, which also includes receptors for corticosteroids, vitamin D_3_, thyroid hormones, and retinoic acids [5]. They are ligand-dependent transcription factors with three characteristic domains that are structurally similar: a transcription activation domain (AD), DNA-binding domain (DBD), and ligand-binding domain (LBD) located from the N to C terminus. Ligand-bound receptors translocate from the cytosol into the nucleus, at which they bind as their homodimers to specific DNA sequences (*cis*-element) called estrogen response elements (EREs) for estrogen receptors [6, 7] and glucocorticoid response elements (GREs) for AR and PR [8, 9]. Following the recruitment of co-regulators that activate or repress NR-dependent transcription, sex steroid hormone receptor complexes temporally and spatially regulate the transcription of target genes on the genome [10–12].

Sex steroid hormones are synthesized not only in the gonads, but also in the adrenal glands and brain. By influencing the reproductive tract, sexual phenotype, and secondary sexual characteristics of male and female animals including humans, sex steroid hormones entirely control the reproductive process: sexual development and maturation, sex-dependent brain differentiation, and sexual behavior [3, 13–15]. These steroids affect other tissues in non-reproductive systems that are not traditionally regarded as targets. For example, estrogens exert protective effects against osteoporosis, neurodegenerative diseases, cardiovascular disease, and obesity [16, 17]. Sex steroid hormones are also involved in the development of cancers, such as breast, ovarian, and endometrial cancers in females and prostate cancer in males [18]. Therefore, these sex steroids are considered to be important clinical tools in diagnostic and prognostic research, and synthetic chemicals with the ligand activities of sex steroid receptors have been developed for therapeutic approaches [19–22].

Natural and synthetic steroid hormones, including sex steroids, excreted into the environment have recently been identified as a serious public health threat due to their adverse effects on humans and wild life [23–25]. Simple reporter gene assay systems based on mammalian cells and budding yeasts have been developed to detect environmental steroid contaminants [26–31]. We and other researchers reported that various chemical substances, including corticosteroids, were detectable at greater sensitivity in yeast strains in which genes encoding the cell wall mannoproteins *CWP1* and *CWP2* and/or plasma membrane-localized ATP-binding cassette (ABC) transporters *PDR5* and *PDR10* were deleted than in wild-type yeast strains [28, 32–35]}. The sensitivities of yeast-based reporter gene assay systems are generally less than those of mammalian cell-based assay systems [26, 31]. In yeasts, the cell wall provides a strong physical barrier to restrict the permeability of chemical substances into cells. Cwp1p and Cwp2p, major mannoproteins, are localized in the external cell wall layer and important for maintaining a rigid wall structure in yeasts [36–38]. In addition, pleiotropic drug resistance (PDR) mediated by plasma membrane-localized ABC transporters, which constitute active drug efflux pumps, functions as a dynamic biochemical xenobiotic defense system in yeasts. Various unrelated chemicals with a broad spectrum are pumped into the extracellular space in order to minimize their toxic effects. Intracellular chemical concentrations are considered to be elevated due to enhanced permeability and/or the inhibition of the extracellular efflux of substances in mutants.

In the present study, we constructed yeast strains that lacked the cell wall mannoproteins Cwp1p/Cwp2p and/or plasma membrane efflux pumps Pdr5p/Pdr10p and expressed the human sex steroid hormone receptors, ERα, ERβ, AR, and PR, to establish highly sensitive reporter gene assays. The ligand-specific responses of these newly established reporter gene assay strains were compared with those of wild-type assay strains using natural and synthetic ligands, pharmaceuticals, and human urine.

## Materials and methods

### Strains and media

The *Escherichia coli* strain, DH5α, was used as a host strain to amplify plasmid DNA. *Saccharomyces cerevisiae* strains are listed in Table S-1. All *S. cerevisiae* strains used in the present study were isogenic with W303**a** (*MAT*a, *ura3–1*, *ade2–1*, *trp1–1*, *leu2–3*, *his3–11*, *15*, *can1–100*). Yeast extract peptone dextrose (YPD) and synthetic dextrose complete dropout (SDC-X) media were prepared as previously described [39]. YEP(Gal) and synthetic galactose complete dropout (SGC-X) media contained 1% or 2% (w/v) galactose instead of dextrose.

### Chemicals

Testosterone (TS; purity: ≥97%), 5α-dihydrotestosterone (DHT; ≥95%), trenbolone acetate (TBA; ≥98%), zeranol (Zer; ≥99%), medroxyprogesterone 17-acetate (MPA; ≥98%), corticosterone (CS; ≥95%), and hydrocortisone (HC; ≥97%) were purchased from Wako Pure Chemical Industries, Ltd. (Osaka, Japan). Dimethyl sulfoxide (DMSO), dithiothreitol (DTT), 17β-estradiol (E2; ≥97%), and progesterone (PS; ≥97%) were obtained from Nacalai Tesque (Kyoto, Japan). Diethylstilbestrol (DES; ≥99%), aldosterone (AS; ≥95%), estrone (E1; ≥99%), estriol (E3; ≥99%), tamoxifen (Tam; ≥99%), 17β-hydroxy-17-methylandrosta-1,4-dien-3-one (17β-H; ≥98%), flutamide (Flu; ≥99%), 17α-methyltestosterone (17α-MTS; ≥98%), mifepristone (Mif; ≥98%), spironolactone (Spi; ≥97%), stanozolol (STA; ≥98%), 19-norethindrone (NET; ≥98%), desogestrel (DSG; ≥98%), drospirenone (DRSP; ≥98%), methyl-piperidino-pyrazoledihydrochloride hydrate (MPP; ≥97%), and *o-*nitrophenyl-β-D-galactopyranoside (ONPG) were purchased from Sigma Aldrich Chemical Co. (St. Louis, MO, USA). Ethinyl estradiol (EE2; ≥98%) was purchased from Cayman Chemical Company (Ann Arbor, MI, USA). 17α-Methylandrostan-17β-ol-3-one (17α-MAS; ≥93%) was obtained from Tokyo Chemical Industry Co., Ltd. (Tokyo, Japan). Raloxifen hydrochloride (Ral; ≥98%) and levonorgestrel (LNG; ≥98%), were purchased from LKT Laboratories, Inc. (St. Paul, MN, USA). ICI 182, 780 (ICI; ≥99%), diarylpropionitrile (DPN; ≥99%), and propyl pyrazole triol (PPT; ≥99%) were purchased from Tocris Bioscience (Bristol, UK). Dienogest (DNG; 98%) and gestodene (GTD; 98%) were supplied from Toronto Research Chemicals Inc. (North York, ON, Canada). Restriction enzymes, DNA modification enzymes, and other chemicals were obtained from Wako Pure Chemical Industries, Ltd., TaKaRa Bio Inc. (Otsu, Japan), or TOYOBO Co. (Osaka, Japan).

### Plasmid construction

AR and PR expression plasmids were constructed for the development of AR and PR ligand reporter gene assays. The primer sequences used in the present study were synthesized by Sigma-Aldrich Japan (Tokyo, Japan) and are listed in Table S-2.

DNA fragments containing human AR complementary DNA (cDNA) (DDBJ/EMBL/GenBank accession number M23263) were obtained by a polymerase chain reaction (PCR) from the plasmid pcDNA3.1-AR [40], with the primers ARfRbBm and ARrXh, which contain a restriction site and/or yeast ribosomal-binding consensus sequence near the initiation codon. PCR was performed with high-fidelity PCR polymerase KOD plus (TOYOBO Co., Ltd.), according to the manufacturer’s instructions. The amplified fragment of approximately 2.8 kb was digested with *Bam*HI and *Xho*I, and cloned into the *Bam*HI-*Sal*I sites of the expression vector pUdp6 [41]. The resultant plasmid was designated as pUdp6AR. Plasmids were isolated and purified using the QIAGEN Mini Prep Kit (Valencia, CA, USA).

Human PR cDNA (NM_00926) was amplified from human uterus total RNA (TaKaRa Bio Inc.) by reverse-transcription PCR (RT-PCR). cDNA was obtained using SuperScript III reverse transcriptase (Invitrogen, Carlsbad, CA, USA). Three overlapping DNA fragments containing PR ORF were amplified by PCR using the KOD-plus PCR kit with three sets of primers, hPR1f and hPR948r, hPR801f and hPR1887r, and hPR1745f and hPR2876r, respectively. Using the three amplified DNA fragments of 1 kb each as templates, fusion PCR was performed with the primers hPRfBg and hPRrXh. The amplified fragment of approximately 2.9 kb was digested with *Bgl*II and *Xho*I, and ligated to the *Bam*HI and *Sal*I sites of pUdp6 to construct pUdp6PR. The plasmids were isolated and purified using the QIAGEN Mini Prep kit. The nucleotide sequences of AR and PR ORFs were confirmed using the ABI DNA sequencer (Applied Biosystems, CA, USA).

### Construction of ERα, ERβ, AR, and PR reporter gene assay yeasts

Yeast transformation was performed using the lithium acetate procedure as previously described [42]. ERα and ERβ assay yeasts established in wild-type W303a were previously reported [27]. To construct ERα and ERβ assay yeasts in mutant strains (see Table S-1) [28], the reporter plasmid pTERE-3z carrying three copies of the ERE: AGGTCAACATGACCT (the half site of ERE is underlined) and the expression plasmid for the human transcriptional coactivator SRC1-e, pESC-Leu-SRC1e [27] were introduced in each mutant strain. A transformant from each strain grown on SDC-TRP/LEU agar medium was isolated and used as a host for subsequent transformation. The ERα expression plasmid 2MpUC6-ERα and ERβ expression plasmid pUCura3ERβ [27] were linearized by *Eco*RV and *Nco*I, respectively, and integrated into the *ura3* locus in the yeast genome by homologous recombination. To construct the AR and PR assay yeasts, the reporter plasmid pYTβ-GRE-R8CS carrying eight copies of the GRE: AGAACAAACTGTTCT [28] and pESC-Leu-SRC1e were introduced into wild-type W303a and mutant strains (Table S-1). The AR expression plasmid pUdp6AR and PR expression plasmid pUdp6PR were linearized by *Eco*RV digestion and introduced into transformants with pYTβ-GRE-R8CS and pESC-Leu-SRC1e. Transformants carrying three plasmids, a reporter plasmid, SRC-1e expression plasmid, and NR expression plasmid, were selected on SCD-TRP/LEU/URA agar plates. NRs and SRC-1e were under the control of the *GAL1, 10* dual directional promoter, and their expression was induced by galactose in media. To construct control strains for the antagonist and inverse agonist assays, the plasmids pESC-LEU-CYCp [28] and YCplac 22 (*TRP1*, *CEN4-ARS1*) [43] were introduced into the W303a, YSA172 (*cwp1*Δ*pdr5*Δ), YSA173 (*cwp1*Δ*cwp2*Δ), and YSA354 (*pdr5*Δ*pdr10*Δ) strains (Table S-1), respectively, and *Eco*RV-linearized pUdp6 was subsequently integrated at the *ura3–1* locus by homologous recombination, as described above. Transformants were selected on SDC-TRP/LEU/URA agar plates. The resultant transformants were designated as CYC W303a, CYC *cwp1*Δ*pdr5*Δ, CYC *cwp1*Δ*cwp2*Δ, and CYC *pdr5*Δ*pdr10*Δ, respectively.

### Sex hormone activity assay using ERα, ERβ, AR, and PR reporter yeasts

The assays for AR and PR were conducted as described previously [28]. Briefly, the yeast strains were pregrown overnight at 30 °C in SDC-TRP/LEU/URA medium containing 2% glucose, and the optical density (OD) at 595 nm of each culture was adjusted to 1.0 with the same medium. A 1-μl aliquot of the test chemicals dissolved in DMSO, 10 μl of the overnight culture yeast, and 90 μl of SGC-TRP/LEU/URA containing 2% galactose were mixed in a 96-well polystyrene microplate with subsequent incubation for 18 h at 30 °C (glucose:galactose = 0.2%:1.8%). In the assays for ERα and ERβ, SDC-TRP/LEU/URA/PHE/TYR medium containing 2% glucose was used for the preculture. Ten-microliter aliquots of the yeast suspension (adjusted OD_595_ to 1.0) were mixed with 1 μl of the test chemical and 90 μl of SGC-TRP/LEU/URA/PHE/TYR containing 1% galactose instead of 2% galactose to induce the expression of ERs and SRC-1e in ligand exposure (glucose:galactose = 0.2%:0.9%). When the agonistic activities of EE2, DES, DPN, and PPT were examined, each yeast culture pregrown in SDC-TRP/LEU/URA/PHE/TYR medium containing 2% glucose was resuspended in synthetic dropout medium (−TRP/LEU/URA/PHE/TYR) containing 1.2% glucose and 0.8% galactose in order to reduce ligand-independent *lacZ* expression. OD_595_ of the suspension was adjusted to 0.1 with the same medium. One microliter of the test chemical was mixed with 100 μl of the yeast cell suspension in 96-well microtiter plates, which were then incubated at 30 °C for 18 h.

Each cell suspension (5 μl for ERα and ERβ, and 10 μl for AR and PR) was transferred to a new 96-well microplate and 100 μl of Z-buffer (60 mM Na_2_HPO_4_, 40 mM NaH_2_PO_4_, 1 mM MgSO_4_, 10 mM KCl, 2 mM DTT, and 0.2% sarcosyl, adjusted to pH 7.0), containing 1 mg/ml ONPG, was added to the plates with subsequent incubation at 37 °C for 60 min. Absorbance at wavelengths of 405 and 595 nm was measured using Micro Plate Reader Model 680 (BioRad Laboratories, Inc.) to estimate β-galactosidase activity as the amount of *o*-nitrophenol produced and yeast cell density, respectively. Agonist-dependent *lacZ* reporter induction was demonstrated as “an increase of induction”, which was calculated using the following formula: [OD_405_ (sample)/OD_595_ (sample)]-[OD_405_ (DMSO)/OD_595_ (DMSO)].

The antagonistic activities of chemicals were examined by competition between the antagonist and agonist added at the concentration of EC_50_. In the antagonist assays for ERs, E2 (0.18 nM and 0.022 nM for the ERα-expressing wild-type and *cwp1*Δ*cwp2*Δ strains, and 0.14 nM and 0.037 nM for the ERβ-expressing wild-type and *cwp1*Δ*cwp2*Δ strains, respectively) was mixed with test ligands. To examine antagonist effects on AR in yeast strains, testosterone (24, 21, and 13 nM for the wild-type, *cwp1*Δ*pdr5*Δ, and *pdr5*Δ*pdr10*Δ strains, respectively) was used as an agonist with test compounds. In the PR antagonist assay, PS (2.31, 0.08, and 0.15 μM for the wild-type, *cwp1*Δ*pdr5*Δ, and *cwp1*Δ*cwp2*Δ strains, respectively) was mixed with test ligands. The dose-dependent repression of *lacZ* reporter expression by antagonists is shown as “relative activity” (%), which was calculated using the following formula: [OD_405_ (sample)/OD_595_ (sample)]/[OD_405_ (DMSO)/OD_595_ (DMSO)] × 100. To distinguish antagonist activity from the non-specific inactivation of gene expression, the CYC yeast strains established in W303a and corresponding mutants for each receptor that constitutively expressed the *lacZ* reporter were used for comparisons.

### Extraction and concentration of synthetic sex hormone activities from oral tablets

To test the sex hormone activities of pharmaceutical products, ten oral tablets of Lunabell® were ground into a fine powder in a mortar and suspended in methanol (1 g/12 ml) in polycarbonate tubes. These tubes were shaken for five minutes to extract organic substances. After centrifugation at 4000 rpm for 5 min, the supernatants were transferred to 1.5-ml microtubes (1 ml/tube) and dried using a vacuum concentrator. Extracts were redissolved in 15 μl of DMSO and used for yeast reporter gene assays.

### Extraction and concentration of organic substances from urine

The human biological sample, urine was provided from two independent donors, an adult male and female. Both donors gave written informed consent. To detect ERα, ERβ, AR, and PR ligand activities, 50 ml of urine from the donors was filtered using a GF/C 47 mmϕ membrane (Whatman International Ltd., Maidstone, England) and acidified by adding H_2_SO_4_ at a final concentration of 0.5 M for deconjugation. After an incubation at 60 °C for 1 h, urine samples were neutralized using 10 N NaOH and flowed through a Waters Sep-pak Plus C18 Environmental cartridge (Waters Corporation, MA, USA). Bound substances were eluted from the cartridge with 2 ml of DMSO at a flow rate of 1 ml/min and then dried using a vacuum concentrator. The extract was redissolved in 500 μl of DMSO (i.e., concentration factor of 100). Samples were serially diluted and used as test samples in the yeast reporter gene assays. Data obtained from samples with the concentration factor of 50 were analyzed using the Student’s *t-*test to assess significance between male and female urine. Probability (*p*) values < 0.01 were considered significant.

## Results

### Response to endogenous ligands of newly constructed sex hormone receptor-expressing yeast strains lacking CWPs and/or ABC transporter genes

**Fig. 1:**
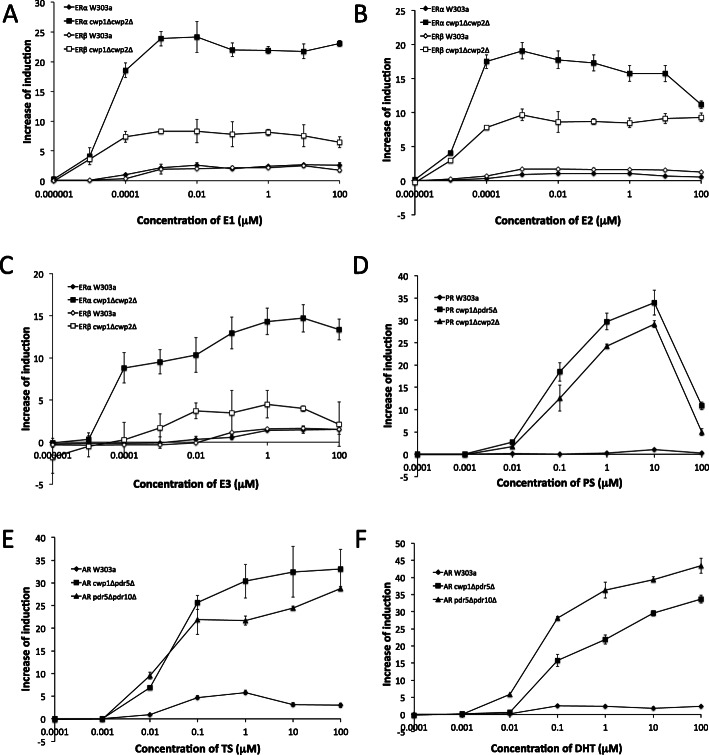
Responses of reporter gene assay yeast strains expressing human ERα, ERβ, PR, and AR to endogenous sex steroid hormones. ERα and ERβ assay yeasts (W303a and *cwp1*Δ*cwp2*Δ) were exposed to E1 (A), E2 (B), and E3 (C). PR (W303a, *cwp1*Δ*pdr5*Δ, and *cwp1*Δ*cwp2*Δ) and AR (W303a, *cwp1*Δ*pdr5*Δ, and *pdr5*Δ*pdr10*Δ) assay yeasts were exposed to PS (D), TS (E), and DHT (F), respectively. The ligand-dependent induction of β-gal was measured as described in the Methods section. Data represent the mean ± SD of triplicate experiments

The reporter gene assay yeast strains expressing human ERα, ERβ, PR, and AR established in wild-type W303a showed the dose-dependent induction of the *lacZ* reporter gene in response to the human endogenous ligands, E2, PS, and TS, respectively (Fig. 1). The EC_50_ values of ERα, ERβ, PR, and AR were 0.18 nM, 0.14 nM, 2.31 μM, and 24.1 nM, respectively (Table 1); however, sensitivities were markedly lower than the reporter gene assay systems in mammalian cells [26]. Since the deletion of genes encoding CWPs (*CWP1*/*CWP2*) and/or ABC transporter efflux pumps (*PDR5/PDR10*) on the plasma membrane enhanced the sensitivities of DNA damage-sensing yeasts and corticosteroid reporter gene assay yeasts [28, 33, 34], we used deletion mutants of these genes as hosts to establish new reporter gene assay strains. As shown in Fig. S1, stronger and more sensitive reporter gene expression was observed in most of the eleven *cwp/pdr* mutants. Based on stronger ligand-dependent and weaker ligand-independent (background) reporter expression, *cwp1*Δ*cwp2*Δ was selected as the most responsive mutant for ERα and ERβ assays; *cwp1*Δ*cwp2*Δ and *cwp1*Δ*pdr5*Δ for PR assays; and *cwp1*Δ*pdr5*Δ and *pdr5*Δ*pdr10*Δ for AR assays (Fig. S1).

**Table 1:** Comparison of responses to endogenous agonist ligands among wild-type and various mutant yeast strains expressing sex steroid hormone receptors

Ligand	E1			
Yeast strain	ERα W303a	ERα *cwp1*Δ*cwp2*Δ	ERβ W303a	ERβ *cwp1*Δ*cwp2*Δ
Fold difference (10^−3^ μM)	1	10.9	1	4.3
Detection limit (μM)	10^−4^	10^−5^	10^− 4^	10^− 5^
EC_50_ (μM)	1.20 × 10^− 4^	2.28 × 10^− 5^	2.51 × 10^− 4^	1.50 × 10^− 5^
Ligand	E2			
Yeast strain	ERα W303a	ERα *cwp1*Δ*cwp2*Δ	ERβ W303a	ERβ *cwp1*Δ*cwp2*Δ
Fold difference (10^−3^ μM)	1	21.7	1	5.7
Detection limit (μM)	10^−4^	10^−5^	10^− 4^	10^− 5^
EC_50_ (μM)	1.84 × 10^− 4^	2.23 × 10^− 5^	1 .42 × 10^− 4^	3.65 × 10^− 5^
Ligand	E3			
Yeast strain	ERα W303a	ERα *cwp1*Δ*cwp2*Δ	ERβ W303a	ERβ *cwp1*Δ*cwp2*Δ
Fold difference (1 μM)	1	10.1	1	2.9
Detection limit (μM)	0.01	10^−5^	0.1	10^−4^
EC_50_ (μM)	0.15	3.02 × 10^−5^	0.034	1.22 × 10^−3^
Ligand	PS			
Yeast strain	PR W303a	PR *cwp1*Δ*pdr5*Δ	PR *cwp1*Δ*cwp2*Δ	
Fold difference (10 μM)	1	34.2	29.4	
Detection limit (μM)	10	0.01	0.01	
EC_50_ (μM)	2.31	0.08	0.15	
Ligand	TS			
Yeast strain	AR W303a	AR *cwp1*Δ*pdr5*Δ	AR *pdr5Δpdr10Δ*	
Fold difference (1 μM)	1	5.2	3.7	
Detection limit (μM)	0.01	0.01	0.01	
EC_50_ (μM)	0.024	0.021	0.013	
Ligand	DHT			
Yeast strain	AR W303a	AR *cwp1*Δ*pdr5*Δ	AR *pdr5Δpdr10Δ*	
Fold difference (100 μM)	1	14.4	18.6	
Detection limit (μM)	0.01	0.01	0.01	
EC_50_ (μM)	0.028	0.011	0.023	

Abbreviation used: EC_50_, 50% effective concentration

Fig. 1 shows the dose-dependent induction of the *lacZ* reporter gene against endogenous ligands in newly-constructed mutant and wild-type yeast strains for the ERα, ERβ, PR, and AR assays. The responses of ERα- and ERβ-expressing *cwp1*Δ*cwp2*Δ strains to the three endogenous estrogens E1, E2, and E3 and PR-expressing *cwp1*Δ*cwp2*Δ or *cwp1*Δ*pdr5*Δ strains to PS were markedly stronger than those of the corresponding receptor-expressing wild-type W303a strains: relative reporter activities markedly increased (3- to 34-fold), while EC_50_ values (4- to 5000-fold) and the minimum detection limit (10- to 1000-fold) markedly decreased (Fig. 1 and Table 1). AR-expressing *cwp1*Δ*pdr5*Δ and *pdr5*Δ*pdr10*Δ strains also showed stronger relative reporter activities than the wild-type W303a strain (4- to 19-fold) (Fig. 1 and Table 1); however, EC_50_ values were only 1.2- to 2.4-fold lower than those of the wild-type strain, and the minimum detection limit was not changed in the mutant and wild-type strains (Table 1). We also compared ligand potencies of endogenous estrogens and androgens in the yeast assay. E1 and E2 were more potent ligands than E3 in ERα- and ERβ-expressing stains. E1 and E2 for ERs and TS and DHT for AR showed similar potencies in yeasts (Fig. 1. and Table 1).

### Cross-reactivity of newly constructed sex hormone receptor assay yeast strains against various steroid hormones

**Fig. 2:**
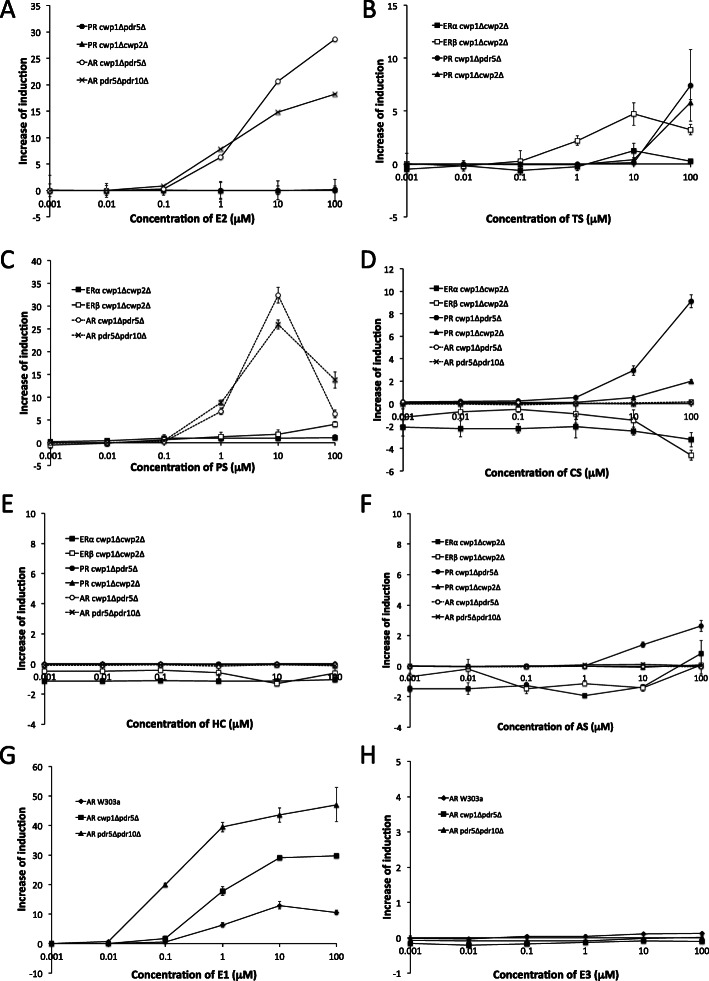
Cross-reactivity of *cwp/pdr* mutant reporter gene assay yeast strains expressing ERα, ERβ, PR, and AR against endogenous steroid hormones. (A-C) Responses to non-primary sex steroid hormones were examined: E2 for AR and PR (A), TS for ERα, ERβ, and PR (B), and PS for ERα, ERβ, and AR (C). (D-F) Responses to corticosteroids: CS (D), HC (E), and AS (F) for ERα, ERβ, PR, and AR. Responses of AR assay yeasts to E1 and E3 were also examined (G and H). The wild-type AR assay yeast was also used as a reference. Data represent the mean ± SD of triplicate experiments

To examine the ligand specificity of each receptor in the reporter yeast strains, we performed a reporter gene assay using sex hormones and corticosteroid hormones as ligands. Although their EC_50_ values were markedly higher than those of intrinsic primary ligands, some cross-activated other receptors and induced *lacZ* reporter gene expression (Table S-3). AR-expressing *cwp1*Δ*pdr5*Δ and *pdr5*Δ*pdr10*Δ mutant strains were activated by E2 and PS at doses higher than 0.1 μM, and maximum reporter activities were similar to those of ERα- and ERβ-expressing mutant strains to E2 and those of PR-expressing mutant strains to PS (Fig. 1 and Fig. 2a, c). The cross-reactivity of AR to E2 and PS was markedly stronger in the mutants than in wild-type W303a (14- to 38-fold stronger reporter activity and 10-fold lower minimum detection limit, data not shown). AR, particularly in *pdr5*Δ*pdr10*Δ, strongly responded to the other natural estrogen E1, but not to E3 (Fig. 2g and h). The ERβ-expressing *cwp1*Δ*cwp2*Δ mutant strain weakly responded to TS and PS at high doses (Fig. 2b and c). PR-expressing *cwp1*Δ*cwp2*Δ and *cwp1*Δ*pdr5*Δ mutant strains exhibited moderate reporter gene expression in response to TS and the corticosteroids, CS and AS, at high doses (Fig. 2b, d-f). ERα-expressing *cwp1*Δ*cwp2*Δ strains did not respond to any steroid hormone other than estrogens (Fig. 2 and Table S-3).

### Detection of agonist activities of synthetic ligands in ERα, ERβ, and AR assay yeast strains

We tested the responses of ERα- and ERβ-expressing yeast strains to the synthetic agonist ligands EE2 and DES, and two selective ER modulators (SERMs), PPT and DPN. EE2 is bioactive and is the most widely used synthetic estrogen for hormone therapy with oral and transdermal administration commonly used worldwide [44, 45]. DES was the first orally active synthetic non-steroidal estrogen prescribed to pregnant women to reduce the risk of abortion between the 1940s to 1970s in Western countries. However, it is now known as a teratogen and carcinogen because of its detrimental effects in humans [46, 47]. PPT activates ERα, but not ERβ, while DPN shows higher selectivity for ERβ than for ERα [48–50].

**Fig. 3:**
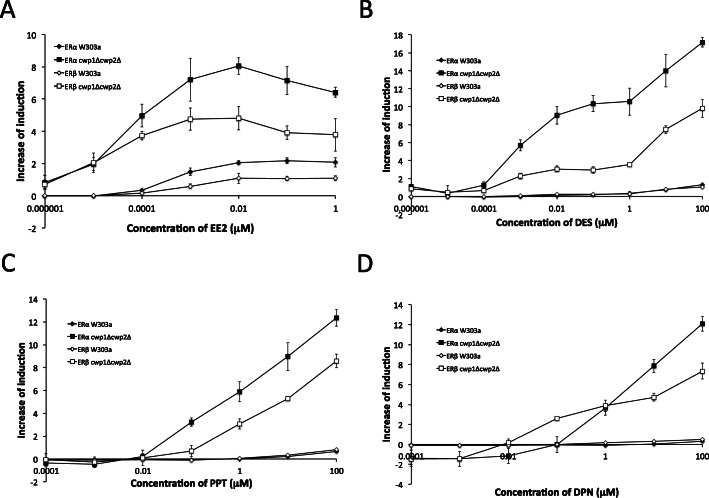
Responses of reporter gene assay yeast strains expressing ERα and ERβ (W303a and *cwp1*Δ*cwp2*Δ) to synthetic estrogens. ERα and ERβ assay yeasts were exposed to EE2 (A), DES (B), PPT (C), and DPN (D). In the assay of these ligands, medium composition was changed from that used for other estrogen assays to reduce ligand-independent β-gal activity (data not shown, see Materials and Methods for details). Data represent the mean ± SD of triplicate experiments

ERα- and ERβ-expressing *cwp1*Δ*cwp2*Δ strains exhibited stronger responses to EE2 with markedly lower EC_50_ values than those of the wild-type W303a strain (Fig. 3a and Table 2). In ERα- and ERβ-expressing W303a, reporter activities induced by DES and two SERMs were very low, even at high doses. The responses of both ERs to these ligands were markedly stronger in *cwp1*Δ*cwp2*Δ, with lower EC_50_ values and detection limit and increased reporter activities (Fig. 3b-d and Table 2). The subtype-selective ligand activity of SERMs was also detectable in the *cwp1*Δ*cwp2*Δ strain. DPN showed selectivity for ERβ as previously reported, with a 35.5- and 10-fold lower EC_50_ value and detection limit, respectively, than ERα. PPT activated not only ERα, but also ERβ: the EC_50_ value and minimum detection limit for ERα were 2.5 and 10-fold lower, respectively, than those for ERβ (Fig. 3c, d and Table 2).

**Table 2:** EC_50_ values (μM) of synthetic ligands in yeast strains expressing human ERα, ERβ, and AR

Receptor	Strains	Ligands					
		E2*	EE2	DES	PPT	DPN	
ERα	W303a	7.66 × 10^−4^	6.34 × 10^−4^	4.20	18.42	26.04	
	*cwp1*Δ*cwp2*Δ	8.34 × 10^−5^	4.22 × 10^−5^	3.91	0.92	3.16	
ERβ	W303a	1.89 × 10^−4^	6.59 × 10^− 4^	8.31 × 10^− 4^	13.71	3.54	
	*cwp1*Δ*cwp2*Δ	5.89 × 10^− 5^	1.76 × 10^− 5^	8.50 × 10^− 4^	2.34	0.089	
Receptor	Strains	Ligands					
		17α-MAS	17β-H	17α -MTS	TBA	STA	EE2
AR	W303a	0.010	0.032	11.6	0.10	0.019	6.47
	*cwp1*Δ*pdr5*Δ	0.027	0.019	0.078	0.031	0.022	2.88
	*pdr5*Δ*pdr10*Δ	3.10 × 10^−3^	0.016	0.023	0.75	0.021	1.88

* Dose-response curve of ERα- and ERβ-expressing yeast strains against E2 assayed in exposure condition for synthetic estrogens was presented in Fig. S2

**Fig. 4:**
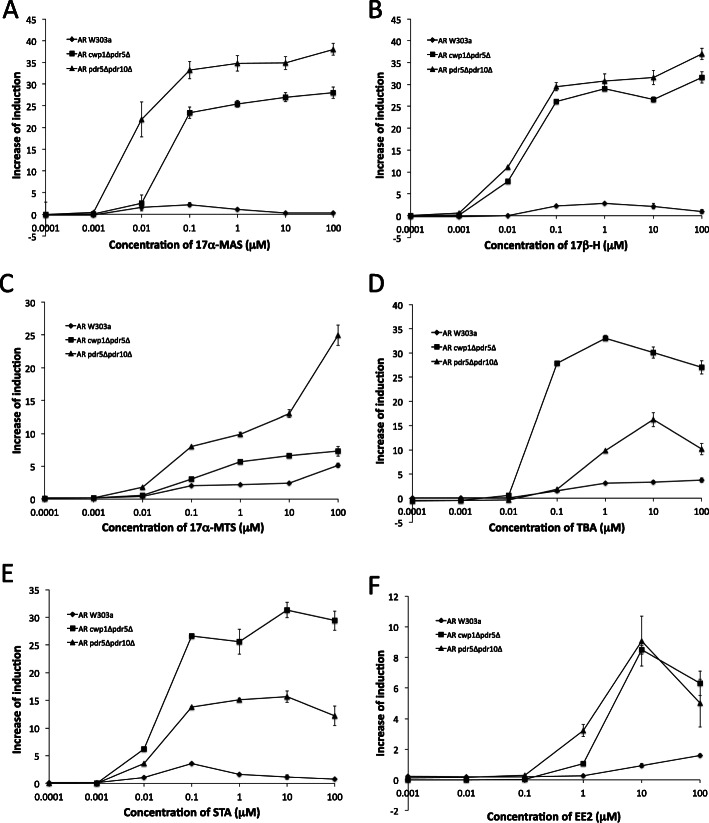
Responses of reporter gene assay yeast strains expressing AR (W303a, *cwp1*Δ*pdr5*Δ, and *pdr5*Δ*pdr10*Δ) to synthetic androgens and EE2. AR assay yeasts were exposed to 17α-MAS (A), 17β-H (B), 17α-MTS (C), TBA (D), STA (E), and EE2 (F). Data represent the mean ± SD of triplicate experiments

We then performed a reporter gene assay to examine the responses of AR-expressing strains to synthetic ligands known as anabolic androgenic steroids [51–55]. As shown in Fig. 4, the mutant strains *cwp1*Δ*pdr5*Δ and *pdr5*Δ*pdr10*Δ expressing AR responded to synthetic ligands more strongly than the wild-type W303a strain. The AR-expressing *cwp1*Δ*pdr5*Δ strain showed stronger responses to TBA and STA than the *pdr5*Δ*pdr10*Δ strain, while the AR-expressing *pdr5*Δ*pdr10*Δ strain was more responsive to 17α-MAS and 17α-MTS than the *cwp1*Δ*pdr5*Δ strain (Fig. 4a-e and Table 2). We also showed that AR strains, particularly mutant strains, detected the synthetic estrogen EE2 (Fig. 4f) as well as the natural estrogens E1 and E2 (Fig. 2a, g and Table 2).

### Responses of estrogen receptor assay yeast strains to known antagonist ligands

**Fig. 5:**
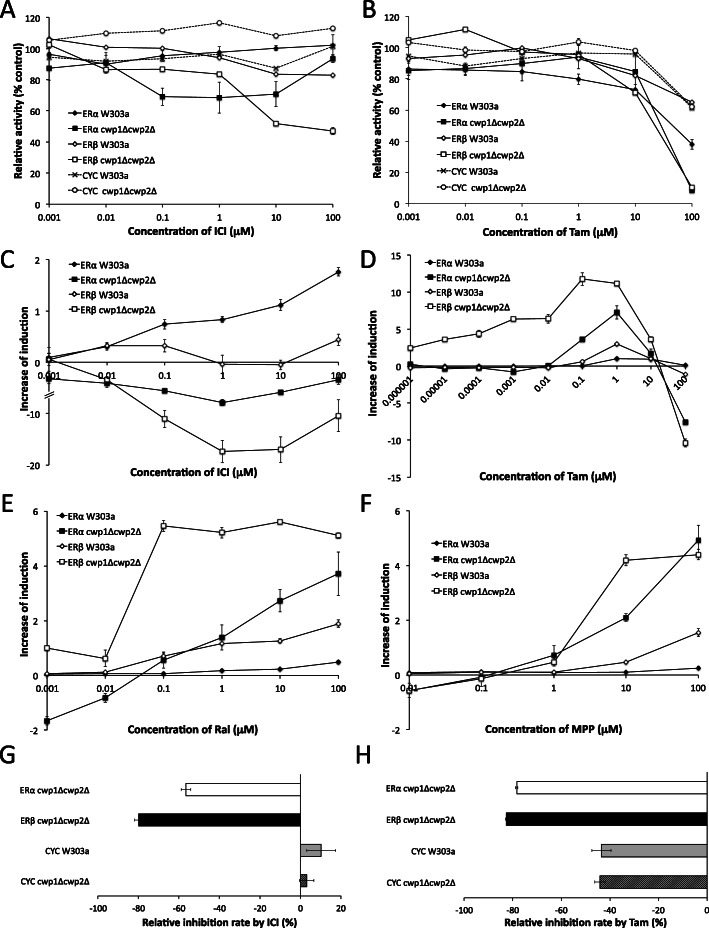
Responses of reporter gene assay yeast strains expressing ERα and ERβ (W303a and *cwp1*Δ*cwp2*Δ) to known antagonist ligands. ERα and ERβ assay yeasts were exposed to ICI (A and C), Tam (B and D), Ral (C), and MPP (F). Antagonistic activity (A and B) and partial agonist activity (C-F) were measured. Regarding ICI and MPP, inverse agonist activity was also measured: ICI (G) and Tam (H). CYC yeasts (W303a and *cwp1*Δ*cwp2*Δ) that constitutively express β-gal were used as references in antagonist and inverse agonist assays. Data represent the mean ± SD of triplicate experiments

We investigated the responses of newly-constructed yeast strains expressing ERα and ERβ to known antagonistic ligands by simultaneously exposing them to the agonist E2: 0.18 nM and 0.022 nM for the ERα-expressing wild-type and *cwp1*Δ*cwp2*Δ strains, and 0.14 nM and 0.037 nM for the ERβ-expressing wild-type and *cwp1*Δ*cwp2*Δ strains, respectively. The strains CYC W303a and CYC *cwp1*Δ*cwp2*Δ, which constitutively express β-galactosidase, were used as references (see Materials and Methods for details). As shown in Fig. 5a, E2-dependent transactivation activity was moderately inhibited by ICI in ERα- and ERβ-expressing *cwp1*Δ*cwp2*Δ strains (Fig. 5a and Table S-4). Constitutive β-gal expression was not repressed by ICI in control CYC strains up to 100 μM (Fig. 5a). In the Tam assay, antagonist activity was detectable in ERα-expressing W303a and *cwp1*Δ*cwp2*Δ, and in ERβ-expressing *cwp1*Δ*cwp2*Δ. At a concentration of 100 μM, the expression of β-gal was almost completely inhibited in ERα- and ERβ-expressing *cwp1*Δ*cwp2*Δ. Although Tam also inhibited the constitutive expression of β-gal in CYC strains at 100 μM, this inhibitory effect was markedly stronger in both ER-expressing yeast strains (Fig. 5b). These results indicate that the antagonistic activity of ICI and Tam was detectable in ERα and ERβ-expressing *cwp1*Δ*cwp2*Δ strains (Fig. 5a, b, and Table S-4). In contrast to ICI and Tam, the antagonistic activity of Ral and MPP was undetectable in the yeast reporter gene assay (data not shown). We also performed an agonist assay using these four chemicals as ligands. ERα- and ERβ-expressing yeast strains dose-dependently detected the partial agonist activities of ICI, Tam, Ral, and MPP. The responses observed were markedly stronger in *cwp1*Δ*cwp2*Δ than in W303a strains (except for the ICI assay). (Fig. 5c-f and Table S-5). However, ERα- and ERβ-expressing *cwp1*Δ*cwp2*Δ strains showed unexpected responses to ICI and Tam. Reporter activity was abolished at some doses (ICI up to 1 μM, and Tam at 100 μM) in agonist assay (Fig. 5c and d). This observation suggests that basal β-gal expression levels due to ligand-independent transactivation activity of ERα and ERβ were inhibited by ICI and Tam.

To clarify whether ERα- and ERβ-expressing *cwp1*Δ*cwp2*Δ strains detected the inverse agonist activity of these chemicals [56, 57], we performed an agonist assay using ERα- and ERβ-expressing *cwp1*Δ*cwp2*Δ strains along with the control CYC strains constitutively expressing β-gal. ICI strongly inhibited basal β-gal expression by ERα and ERβ in *cwp1*Δ*cwp2*Δ at 1 μM, while constitutive β-gal activity was not affected in CYC strains (Fig. 5g). When Tam was exposed at 100 μM, constitutive β-gal expression in control CYC yeasts was reduced to less than 60%, showing cytotoxic effects. However, basal β-gal levels were strongly inhibited in ERα- and ERβ-expressing *cwp1*Δ*cwp2*Δ strains: residual β-gal activity was ~ 20% (Fig. 5h). These results indicated that the inverse agonist activity of ICI and Tam on ERs was detectable in the yeast reporter gene assay.

### Examination of ligand activities of Mif and Spi for PR, and flu and Zer for AR in yeast reporter gene assays

**Fig. 6:**
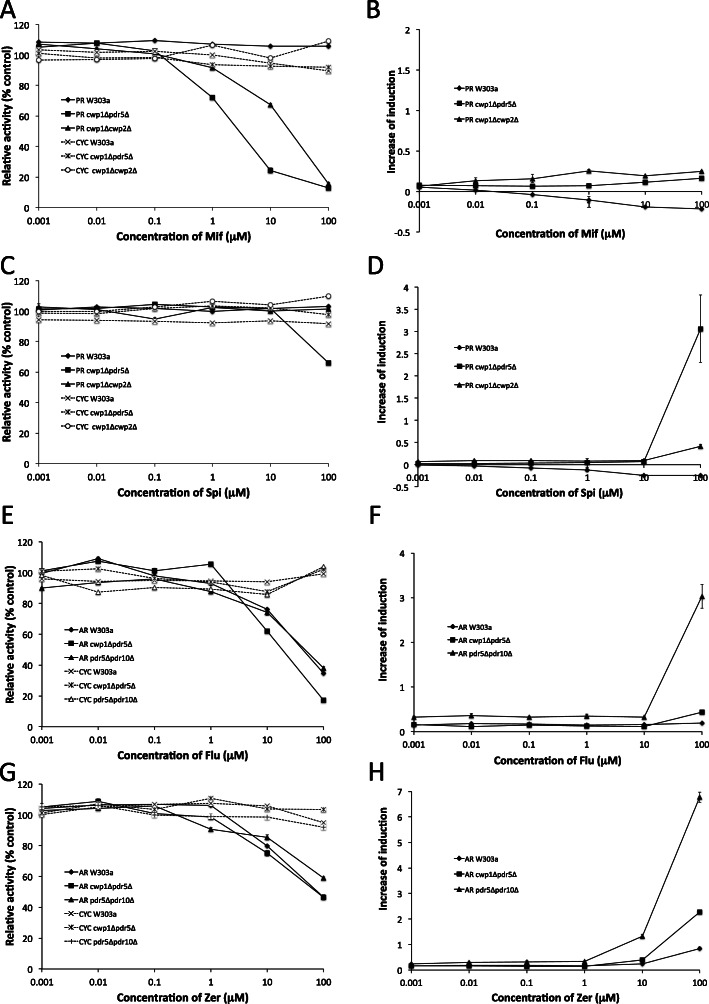
Responses of reporter gene assay yeast strains expressing PR (W303a, *cwp1*Δ*pdr5*Δ, and *cwp1*Δ*cwp2*Δ) and AR (W303a, *cwp1*Δ*pdr5*Δ, and *pdr5*Δ*pdr10*Δ) to known antagonist ligands. PR assay yeasts were exposed to Mif (A and B) and Spi (C and D), and AR assay yeasts were exposed to Flu (E and F) and Zer (G and H). Antagonistic activity (A, C, E, and G) and partial agonist activity (B, D, F, and H) were measured. CYC yeasts (W303a, *cwp1*Δ*pdr5*Δ, *cwp1*Δ*cwp2*Δ, and *pdr5*Δ*pdr10*Δ) were used as references in antagonist assays. Data represent the mean ± SD of triplicate experiments

We examined the antagonist and partial agonist activities of Mif and Spi as well as Flu and Zer in the PR- and AR-expressing yeast strains, respectively (Fig. 6 and Table S-4 and S-5). The antagonist assay was performed by co-exposing test compounds with agonist ligands: PS for PR-expressing assay yeasts (2.31, 0.08, and 0.15 μM for the wild-type, *cwp1*Δ*pdr5*Δ, and *cwp1*Δ*cwp2*Δ strains, respectively) and TS for AR assay yeasts (24, 21, and 13 nM for the wild-type, *cwp1*Δ*pdr5*Δ, and *pdr5*Δ*pdr10*Δ strains, respectively), as described above. The constitutive expression of β-gal was not affected by these ligands in the reference CYC strains, CYC W303a, CYC *cwp1*Δ*pdr5*Δ, CYC *cwp1*Δ*cwp2*Δ, and CYC *pdr5*Δ*pdr10*Δ (Fig. 6a, c, e, and g). Mif, a known anti-PS compound with anti-glucocorticoid activity [58] effectively inhibited PS-induced β-gal expression in PR-expressing *cwp1*Δ*cwp2*Δ and *cwp1*Δ*pdr5*Δ strains. Spi, an antagonist ligand for MR and GR [26, 28, 58] with weak progestational and anti-androgenic activities slightly inhibited PS-dependent β-gal expression in the PR-expressing *cwp1*Δ*pdr5*Δ strain (Fig. 6a and c, and Table S-4). In the agonist assay, the PR-expressing *cwp1*Δ*pdr5*Δ strain weakly responded to Spi at 100 μM, but not to Mif (Fig. 6b and d, and Table S-5).

The activities of the known anti-androgenic ligands Flu and Zer were examined in AR assay yeast strains (Fig. 6e-h, and Table S-4). Flu is a specific antagonist for AR [59]. Zer is an anabolic estrogen produced in mycota (mycoestrogen), which is employed for livestock breeding and undescended testicles. Zer is a strong estrogenic metabolite derived from zearalenone, a non-steroidal mycoestrogen produced by *Fusarium* that induces reproductive disorders in domestic animals with estrogenic and anti-androgenic activities [60]. Both chemicals exerted inhibitory effects on TS-induced β-gal activity in a dose-dependent manner (Fig. 6e and g). However, responses to these ligands did not improve as expected in the *cwp1*Δ*pdr5*Δ and *pdr5*Δ*pdr10*Δ strains (Table S-4). AR-expressing *pdr5*Δ*pdr10*Δ strains weakly responded to Flu and Zer at 100 μM in the agonist assay (Fig. 6f and h, and Table S-5).

### Detection of progestational, androgenic, and anti-androgenic activities of progestins in various drug developmental generations

Progestins, a class of synthetic PS, have been used as pharmaceuticals for birth control and the treatment of endometriosis. However, their androgenic activity may cause adverse side effect that is associated with change of lipid metabolism, in particular decrease of high-density lipoprotein cholesterol (HDL) levels and increased risks of cardiovascular diseases [61]. Progestins developed in later generations were less androgenic. Moreover, fourth generation progestins acquired anti-androgenic activity [62, 63]. We tested seven progestins from various generations for the detection of progestational and androgenic activities in PR- and AR-expressing yeast strains: MPA (unclassified), NET (first generation), LNG (second generation), DSG and GTD (third generation), and DNG and DRSP (fourth generation).

**Fig. 7:**
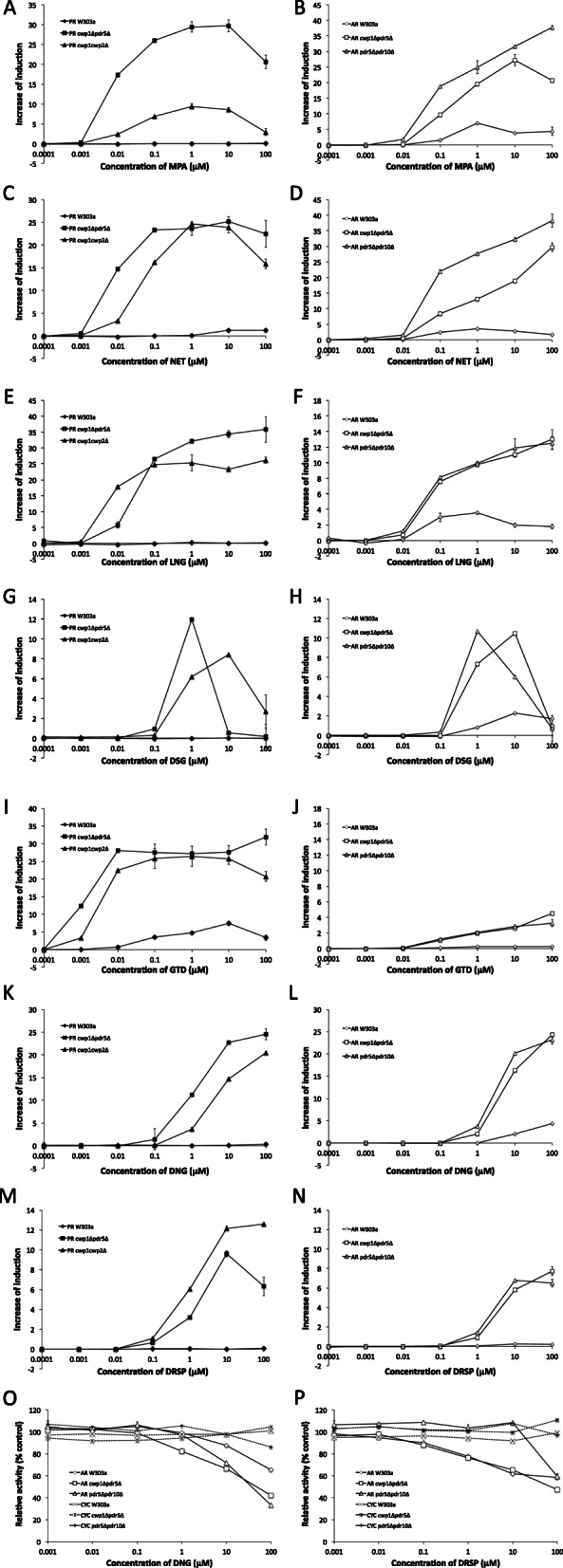
Responses of reporter gene assay yeast strains expressing PR (W303a, *cwp1*Δ*pdr5*Δ, and *cwp1*Δ*cwp2*Δ) and AR (W303a, *cwp1*Δ*pdr5*Δ, and *pdr5*Δ*pdr10*Δ) against progestins from various generations. PR and AR assay yeasts were exposed to MPA (A and B), NET (C and D), LNG (E and F), DSG (G and H), GTD (I and J), DNG (K and L), and DRSP (M and N). Progestational activity (A, C, E, G, I, K, and M) and androgenic activity (B, D, F, H, J, L, and N) were measured. The anti-androgenic activities of DNG (O) and DSRP (P) were also measured by antagonist assays using AR assay yeasts. CYC yeasts (W303a, *cwp1*Δ*pdr5*Δ, and *pdr5*Δ*pdr10*Δ) were used as references. Data represent the mean ± SD of triplicate experiments

In the agonist assay, the responses of the PR-expressing W303a strain to MPA, LNG, DSG, DNG, and DRSP, and those of the AR-expressing W303a strain to GTD and DRSP were weak. The PR- and AR-expressing mutant yeast strains exhibited markedly stronger responses to all seven progestins (Fig. 7a-n). PR agonist activity was more potent than AR agonist activity for the progestins, except DSG (Table 3). A high dose of DSG markedly reduced reporter activity due to cytotoxicity in mutant assay yeast strains (Fig. 7g and h). In comparisons among generations of progestins, PR-expressing mutant yeast strains more strongly responded to the third generation progestin GTD than the earlier generation progestins, MPA, NET, and LNG. Another third generation progestin DSG and the fourth generation progestins DNG and DRSP exhibited markedly weaker agonist potencies for PR than other progestins (Fig. 7a, c, e, g, i, k and m). The PR-expressing *cwp1*Δ*pdr5*Δ strain was more responsive to MPA, NET, GTD, and DNG than the *cwp1*Δ*cwp2*Δ strain. The responses of AR-expressing mutant yeast strains showed that the androgenic activities of the third generation progestins DSG and GTD and fourth generation progestins DNG and DRSP were 10- and 100-fold weaker, respectively, than earlier generation progestins with respect to the minimal detection limit concentration (Fig. 7b, d, f, h, j, l, and n).

**Table 3:** EC_50_ values (μM) of progestins in yeast strains expressing human PR and AR

Receptor	Strains	Ligands						
		MPA	NET	LNG	DSG	GTD	DNG	DRSP
PR	W303a	n. d.	2.92	n. d.	n. d.	0.22	n. c.	n. d.
	*cwp1*Δ*pdr5*Δ	9.70 × 10^− 3^	9.51 × 10^− 3^	0.023	0.29	9.96 × 10^−4^	0.86	1.78
	*cwp1*Δ*cwp2*Δ	0.44	0.07	3.06 × 10^− 4^	0.30	2.59 × 10^− 3^	6.1	0.80
AR	W303a	0.23	0.093	0.030	1.65	n. c.	9.78	n. c.
	*cwp1*Δ*pdr5*Δ	0.28	0.16	0.028	0.32	1.06	8.10	7.39
	*pdr5*Δ*pdr10*Δ	0.084	0.087	0.026	0.31	0.40	2.43	2.32

Abbreviation used: n. d. not detectable, n. c. not calculable

We also performed an antagonist assay to detect the anti-androgenic activities of the fourth generation progestins DNG and DRSP using AR-expressing strains and reference CYC strains. Neither DNG nor DRSP inhibited the constitutive expression of β-gal in CYC strains. In contrast, the dose-dependent inhibition of TS-induced β-gal activity was observed in AR-expressing yeast strains exposed to these compounds. Sensitivity for DNG was improved in mutant assay yeasts expressing AR, while the response to DRSP was similar between the wild-type W303a and mutant assay yeast strains (Fig. 7o and p, and Table S-6).

### Validation of newly constructed sex steroid hormone receptor assay yeast strains

**Fig. 8:**
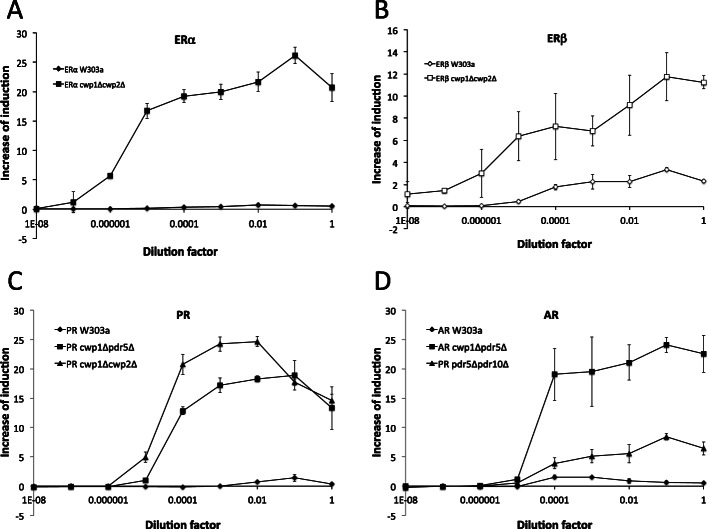
Detection of synthetic sex hormone activities from oral tablets. Organic compounds were extracted from oral pharmaceutical tablets Lunabell® and concentrated. Using serially diluted samples as ligands, yeast reporter gene assays were performed using yeasts expressing ERα and ERβ (W303a and *cwp1*Δ*cwp2*Δ), PR (W303a, *cwp1*Δ*pdr5*Δ, and *cwp1*Δ*cwp2*Δ), and AR (W303a, *cwp1*Δ*pdr5*Δ, and *pdr5*Δ*pdr10*Δ): ERα (A), ERβ (B), PR (C), and AR (D). Data represent the mean ± SD of triplicate experiments

We investigated whether steroid hormone activities were detectable from oral pharmaceutical tablets and human urine samples to validate the newly established reporter gene assay strains for human sex steroid receptors. Organic compounds were extracted and concentrated from the oral tablets and urine containing synthetic and endogenous sex hormones, respectively, and reporter gene assays were performed using the prepared samples as ligands. Lunabell® was developed for the treatment of dysmenorrhea associated with endometriosis. It contains EE2 and NET as pharmaceutically active ligands. As shown in Fig. 8, the assay strains established in wild-type W303a barely detected hormone activities from Lunabell®, except for the ERβ-expressing strain. The mutant yeast strains for all four sex hormone receptors detected ligand activities from the tablets, with a more than 10-fold lower detection limit.

**Fig. 9:**
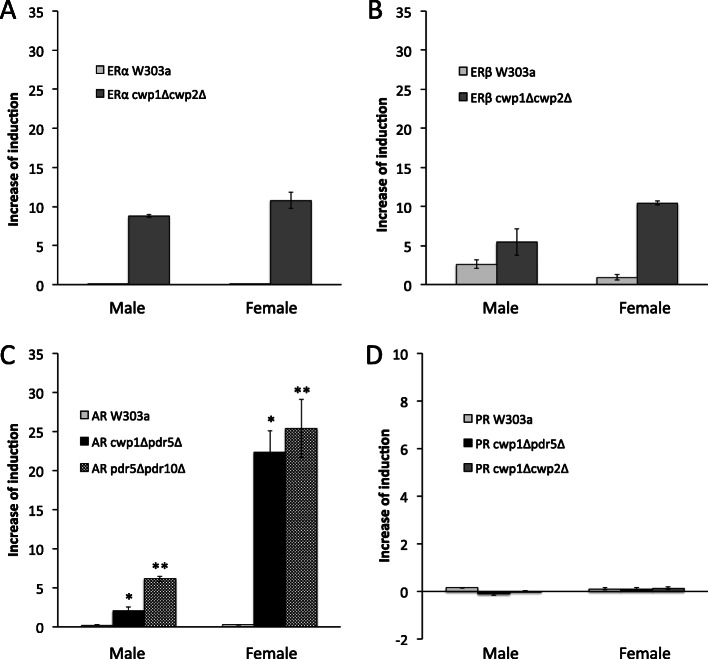
Detection of sex hormone activities from human urine. Organic compounds were extracted and concentrated from urine provided by a male and a female. Samples were serially diluted and used in the reporter gene assay using the yeast strains expressing ERα and ERβ (W303a and *cwp1*Δ*cwp2*Δ), PR (W303a, *cwp1*Δ*pdr5*Δ, and *cwp1*Δ*cwp2*Δ), and AR (W303a, *cwp1*Δ*pdr5*Δ, and *pdr5*Δ*pdr10*Δ): ERα (A), ERβ (B), AR (C), and PR (D). Graphs indicate reporter activity of urine samples with the concentration factor of 50. Data represent the mean ± SD of triplicate experiments. Reporter activity was significantly different between male and female urine samples in AR-expressing mutant strains: *cwp1Δ*pdr5*Δ (*p* < 0.0005); **pdr5Δ*pdr10*Δ (*p* < 0.001)

We then examined whether sex hormone activities in human urine were detectable in the yeast reporter gene assays. The ERβ-expressing wild-type W303a strain weakly responded, and the ERα- and AR-expressing W303a strain barely detected ligand activities in urine. The mutant assay strains expressing these receptors clearly showed reporter activities. None of the PR assay strains responded to urine samples (Fig. 9). In comparisons of male and female urine samples, ERα- and ERβ-expressing *cwp1*Δ*cwp2*Δ strains were slightly more responsive to female urine than to male samples (~ 1.5-fold). In contrast, AR-expressing *cwp1*Δ*pdr5*Δ and *pdr5*Δ*odr10*Δ strains showed markedly stronger responses to female samples than to male samples (Fig. 9). Reporter activity between male and female urine samples were significantly different in AR-expressing *cwp1*Δ*pdr5*Δ (*p* < 0.0005) and *pdr5*Δ*odr10*Δ (*p* < 0.001) strains.

## Discussion

*S. cerevisiae* is the simplest eukaryotic organism that possesses highly conserved gene expression systems with those of higher eukaryotes, including humans [64]. The mechanisms of action of NRs in animal cells may be reconstituted in yeast cells by introducing NRs and transcriptional coactivator genes, along with reporter plasmids containing appropriate response elements [27, 28, 41, 65–69]. In the present study, we established new reporter gene assay systems for human AR and PR and improved our previous ERα and ERβ reporter gene assay yeasts by deleting the *CWP1/CWP2* and/or *PDR5/PDR10* genes. The elimination of the CWPs Cwp1p/Cwp2p and/or plasma membrane efflux pumps Pdr5p/Pdr10p in host yeast cells markedly increased the sensitivities of sex steroid hormone receptors, as observed for corticosteroid receptors in our previous study [28].

The examination of a number of endogenous and synthetic ligands revealed that EC_50_ values and the minimum detection limit of the *cwp*/*pdr* assay yeasts were lower than those of wild-type assay yeast. The only exception was the AR-expressing yeast strain, the sensitivity of which did not markedly differ from that of the wild-type strain (Fig. 1~Fig. 7, Table 1-3, and Table S-3-S-6). Non-polar and hydrophobic steroid compounds may have very low permeability in the yeast cell membrane. To further improve AR-expressing yeast, the deletion of the *ERG6* gene, which is involved in the synthesis of fungal-specific membrane lipid ergosterol, may effectively increase the permeability of the cell membrane [70, 71].

In the assay of ERα- and ERβ-expressing *cwp*/*pdr* mutant yeasts, ERα exhibited markedly stronger reporter activity than ERβ in response to both natural and synthetic estrogenic agonists (Fig. 1a-c, and Fig. 3). This result is consistent with previous findings showing that the transactivation activity of ERβ was weaker than ERα in most cell systems [72, 73], suggesting subtype-specific responses to the effects of estrogen [74–76].

In assays on cross-reactions of steroid hormones with their sex steroid hormone receptors, E1, E2, and PS induced strong AR-mediated reporter gene expression. Although the EC_50_ values of estrogens and PS in the AR reporter gene assay were markedly higher than those of TS and DHT (Table S-3), they induced strong transactivation activity of AR (Fig. 2a and c). In the assay of TS in ERβ and PR-expressing yeasts and CS in PR assay yeasts, ligand responses were weak (Fig. 2b, d, and f). These results correlated with the structural similarity of ligands and LBD among receptors [77–79]. The cross-reactivity observed in the present study reflected the intrinsic potential of ligand-receptor interactions in vivo, which is often associated with the adverse side effects of pharmaceuticals with steroid hormone activity [62, 63, 80]. Cross-reactivity also provides important information for predicting the constituents of sex steroid hormones in reporter gene assays of environmental test samples.

Several unique characteristics of ligand responses were identified in the present study. The ligand potencies of endogenous ligands E1 and E2 for ERα/ERβ and TS and DHT for AR were similar in the yeast assays (Table 1), which is in contrast to previous findings: E2 is more potent ligand than E1 for ERs, and DHT is more potent than TS for AR, respectively, in mammalian cell-based reporter gene assays [26, 49, 81, 82]. In the mammalian cell reporter gene assay, Ral only exhibited partial agonist activity on ERα, but induced reporter activity in both the ERα and ERβ assay yeasts, with stronger responses in ERβ. The partial agonist potential of MPP was observed for both ERα and ERβ (Fig. 5e and f), which has not been reported previously [26]. The antagonism of Ral and MPP was not observed in yeasts, which may have been due to the markedly stronger ligand-independent β-gal activity of ERα- and ERβ-expressing yeasts (data not shown). We also identified an inverse agonist activity of ICI (Fig. 5g), as reported previously in vivo [56]. We detected the moderate inverse agonist activity of Tam on ERα and ERβ (Fig. 5h), suggesting its potential as an inverse agonist on ERs in vivo [57]. Other unique ligand responses in yeasts were observed in the assay of PR and AR against progestins (Fig. 7). Previous studies reported that progestins later than the third drug developmental generation exhibited stronger progestogenic activities and weaker androgenic activities than the previous generations in vivo [62, 63, 83]; however, the sensitivity of PR-expressing assay yeast against DSG, DNG, and DRSP was markedly lower than those of the earlier generations (Fig. 7). The physiological activities of ligands in vivo may be affected by the metabolism of ligands as well as the expression and selectivity of transcriptional coactivators [84–86]. Some ligand substances may be more stable in yeast cells than in mammalian cells due to the lack of metabolic pathways of the ligands, and, thus, substance-receptor interaction potentials may be directly reflected in the yeast reporter gene assay.

Our newly established sex hormone receptor ligand assay yeasts were validated using pharmaceutical tablets and human urine. Lunabell® contains the synthetic estrogen EE2 and first generation progestin NET. *cwp*/*pdr* mutant assay yeasts effectively detected ligand activity contained in the tablets (Fig. 8). Figure 3a and Fig. 7c and d showed that the mutant ERα and ERβ assay strains responded to EE2, while the mutant PR and AR assay strains responded to NET. The mutant AR assay strains also responded to EE2 (Fig. 4f). In the assay of urine, *cwp*/*pdr* mutant assay yeasts expressing ERα and ERβ detected hormone activity excreted into urine from both male and female (Fig. 9a and b). A small amount of estrogens are also produced in males through the conversion of androgens [87]. In contrast, even *cwp*/*pdr* mutant PR yeasts did not detect ligand activity (Fig. 9d). This result suggested that the primary ligand PS or cross-reacting ligand androgens did not exist as active hormone forms in urine [88, 89]. The AR-expressing *cwp*/*pdr* mutant strains strongly detected ligand activity in female sample (Fig. 9c); however, active androgens did not appear to be abundant in female urine. As described above, estrogens E1 and E2 strongly cross-reacted with AR in the *cwp*/*pdr* mutant strains (Fig. 2a and g). The stronger reporter activity observed in mutant AR-expressing yeasts was due to active estrogens contained in female urine.

## Conclusion

In the present study, we newly constructed highly sensitive reporter gene assay yeasts for the sex steroid hormone receptors ERα, ERβ, AR, and PR. Yeast reporter gene assay systems for all six steroid hormone receptors are now available, including previously established GR and MR assay yeasts [28]. By arraying these assay yeasts in a 96-well microtiter plate, environmental endocrine disrupters with steroid hormone activity will be qualitatively and simultaneously detectable by simple and easy procedures. The yeast-based reporter gene assay will be valuable as a primary screening tool to detect and evaluate steroid hormone activities in various test samples. Our assay system will strongly support the detection of agonists, antagonists, and inverse agonists of steroid hormone receptors in the field of novel drug discovery and assessments of environmental pollutants.

## Supplementary information

**Additional file 1: Table S-1.***S. cerevisiae* strains. **Table S-2.** Primer sequences. **Table S-3.** EC_50_ values (μM) of various steroid hormones in yeast strains expressing human sex hormone receptors. **Table S-4.** Comparison of responses of yeast strains expressing human sex hormone receptors against known antagonist ligands. **Table S-5.** Partial agonist activity of known antagonist ligands in yeast strains expressing human sex hormone receptors. **Table S-6.** Anti-AR activity of the fourth generation of progestins in yeast.

**Additional file 2: Figure S1.** Dose-dependent responses to sex steroid hormones in ER, ER, PR, and AR in wild-type W303a and mutants. The reporter assay yeast strains expressing human ER (A), ER (B), PR (C), and AR (D) were established in wild-type yeast W303a and deletion mutants for cell wall mannoproteins (*CWP1*, *CWP2*) and/or plasma membrane efflux pumps (*PDR5*, *PDR10*). The yeast strains were exposed to E2 (A and B), PS (C), and TS (D), and the ligand-dependent induction of -galactosidase activity was measured. **Figure S2.** Dose-dependent responses of ER- and ER-expressing yeast strains exposed to E2 in medium containing increased amounts of glucose. ER- and ER-expressing wild-type W303a and *cwp1cwp2*strains were exposed to E2 in medium containing 1.2% glucose and 0.8% galactose, which was applied for the assay of synthetic estrogens shown in Fig. 3, in order to minimize ligand-independent reporter induction.

## Data Availability

Not applicable.

## Abbreviations

17α-MAS: 17α-Methylandrostan-17β-ol-3-one
17α-MTS: 17α-Methyltestosterone,
17β-H: 17β-Hydroxy-17-methylandrosta-1,4-dien-3-one
AR: Androgen receptor
AS: Aldosterone
CS: Corticosterone
CWP: Cell wall mannoprotein
DES: Diethylstilbestrol
DHT: 5α-Dihydrotestosterone
DMSO: Dimethyl sulfoxide
DNG: Dienogest
DPN: Diarylpropionitrile
DRSP: Drospirenone
DSG: Desogestrel
DTT: Dithiothreitol
E1: Estrone
E2: 17β-Estradiol
E3: Estriol
EE2: Ethinyl estradiol
ER: Estrogen receptor
ERE: Estrogen response element
Flu: Flutamide
GR: Glucocorticoid receptor
GRE: Glucocorticoid response element
GTD: Gestodene
HC: Hydrocortisone
ICI: ICI 182, 780
LNG: Levonorgestrel
Mif: Mifepristone
MPA: Medroxyprogesterone 17-acetate
MPP: Methyl-piperidino-pyrazole dihydrochloride hydrate
MR: Mineralocorticoid receptor
NET: 19-norethindrone
NR: Nuclear receptor
ONPG: *o-*nitrophenyl-β-D-galactopyranoside
PDR: Pleiotropic drug resistance
PPT: Propyl pyrazole triol
PR: Progesterone receptor
PS: Progesterone
Ral: Raloxifen
Spi: Spironolactone
STA: Stanozolol
Tam: Tamoxifen
TBA: Trenbolone acetate
Zer: Zeranole
