# Supplementary material for: Construction of reporter gene assays using CWP and PDR mutant yeasts for enhanced detection of various sex steroids
Source: Genes Environ. 2020 May 27;42:20. doi: 10.1186/s41021-020-00159-x (PMC7251871; doi:10.1186/s41021-020-00159-x)
Supplement: Supplementary file 1 — Additional file 1: Table S-1.S. cerevisiae strains. Table S-2. Primer sequences. Table S-3. EC50 values (μM) of various steroid hormones in yeast strains expressing human sex hormone receptors. Table S-4. Comparison of responses of yeast strains expressing human sex hormone receptors against known antagonist ligands. Table S-5. Partial agonist activity of known antagonist ligands in yeast strains expressing human sex hormone receptors. Table S-6. Anti-AR activity of the fourth generation of progestins in yeast. [file 41021_2020_159_MOESM1_ESM.docx]

**Table S-1** *S. cerevisiae* strains

| Strain | Genotype | Reference |
| --- | --- | --- |
| W303a | *MATa*, *ura3-1*, *ade2-1*, *trp1-1*, *leu2-3*, *his3-11,15*, *can1-100* | Laboratory stock |
| YSA162 | *MATa*, *ura3-1*, *ade2-1*, *trp1-1*, *leu2-3*, *his3-11,15*, *can1-100*, *cwp1*Δ:*loxP* | [28] |
| YSA164 | *MATa*, *ura3-1*, *ade2-1*, *trp1-1*, *leu2-3*, *his3-11,15*, *can1-100*, *cwp2*Δ:*loxP* | [28] |
| YSA167 | *MATa*, *ura3-1*, *ade2-1*, *trp1-1*, *leu2-3*, *his3-11,15*, *can1-100*, *pdr5*Δ:*loxP* | [28] |
| YSA172 | *MATa*, *ura3-1*, *ade2-1*, *trp1-1*, *leu2-3*, *his3-11,15*, *can1-100*, *cwp1*Δ:*loxP*, *pdr5*Δ:*loxP* | [28] |
| YSA173 | *MATa*, *ura3-1*, *ade2-1*, *trp1-1*, *leu2-3*, *his3-11,15*, *can1-100*, *cwp1*Δ:*loxP*, *cwp2*Δ:*loxP* | [28] |
| YSA195 | *MATa*, *ura3-1*, *ade2-1*, *trp1-1*, *leu2-3*, *his3-11,15*, *can1-100*, *cwp2*Δ:*loxP*, *pdr5*Δ:*loxP* | [28] |
| YSA220 | *MATa*, *ura3-1*, *ade2-1*, *trp1-1*, *leu2-3*, *his3-11,15*, *can1-100*, *cwp1*Δ:*loxP*, *cwp2*Δ:*loxP*, *pdr5*Δ:*loxP* | [28] |
| YSA347 | *MATa*, *ura3-1*, *ade2-1*, *trp1-1*, *leu2-3*, *his3-11,15*, *can1-100*, *pdr10*Δ:*loxP* | [28] |
| YSA354 | *MATa*, *ura3-1*, *ade2-1*, *trp1-1*, *leu2-3*, *his3-11,15*, *can1-100*, *pdr5*Δ:*loxP*, *pdr10*Δ:*loxP* | [28] |
| YSA355 | *MATa*, *ura3-1*, *ade2-1*, *trp1-1*, *leu2-3*, *his3-11,15*, *can1-100*, *cwp1*Δ:*loxP*, *pdr10*Δ:*loxP* | [28] |
| YSA357 | *MATa*, *ura3-1*, *ade2-1*, *trp1-1*, *leu2-3*, *his3-11,15*, *can1-100*, *cwp1*Δ:*loxP*, *pdr5*Δ:*loxP*, *pdr10*Δ:*loxP* | [28] |

**Table S-2** Primer sequences

| Primer | Sequence (5’-3’) | Comments |
| --- | --- | --- |
| ARfRbBm | TCGAGGATCCAACAAAATGGAAGTGCAGTTAGGGCTGGG | Amplify AR ORF |
| ARrXh | AGGGGGCTCGAGCTGGGGTGGGGAAATAGG | Amplify AR ORF |
| hPR1f | ATGACTGAGCTGAAGGCAAAGG | Amplify PR ORF |
| hPR801f | GGTCCCCAAGGAAGATTCC | Amplify PR ORF |
| hPR948r | CAATAAGGCGTGATTGAGAGGC | Amplify PR ORF |
| hPR1745f | TACCTGTGGGAGCTGTAAGGT | Amplify PR ORF |
| hPR1887r | CTGACAGCACTTTCTAAGGC | Amplify PR ORF |
| hPR2876r | CAAGACCTCATAATCCTGACC | Amplify PR ORF |
| hPRfBg | GGGCAGATCTACAAAATGACTGAGCTGAAGGCAAA | Amplify PR ORF |
| hPRrXh | CAAGACCTCGAGATCCTGACCAAAC | Amplify PR ORF |

| Receptor | Strains | Ligands | | | | | | | |
| --- | --- | --- | --- | --- | --- | --- | --- | --- | --- |
|  |  | E2 | TS | PS | CS | HC | AS | E1 | E3 |
| ERα | W303a | (1.84 × 10^-4^) | n. d. | n. d. | n. d. | n. d. | n. d. | (1.20 × 10^-4^) | (0.15) |
|  | *cwp1*Δ*cwp2*Δ | (2.23 × 10^-5^) | n. d. | n. d. | n. d. | n. d. | n. d. | (2.28 × 10^-5^) | (3.02 × 10^-5^) |
| ERβ | W303a | (1.42 × 10^-4^) | n. d. | n. d. | n. d. | n. d. | n. d. | (2.51 × 10^-4^) | (0.034) |
|  | *cwp1*Δ*cwp2*Δ | (3.65 × 10^-5^) | 0.87 | 12.0 | n. d. | n. d. | n. d. | (1.05 × 10^-5^) | (1.22 × 10^-3^) |
| PR | W303a | n. d. | n. d. | (2.31) | n. d. | n. d. | n. d. |  |  |
|  | *cwp1*Δ*pdr5*Δ | n. d. | 31.0 | (0.080) | 18.3 | n. d. | 9.7 |  |  |
|  | *cwp1*Δ*cwp2*Δ | n. d. | 29.0 | (0.084) | 20.1 | n. d. | n. d. |  |  |
| AR | W303a | n. c. | (0.03) | 1.57 | n. d. | n. d. | n. d. | 0.90 | n. d. |
|  | *cwp1*Δ*pdr5*Δ | 0.97 | (0.021) | 2.30 | n. d. | n. d. | n. d. | 0.86 | n. d. |
|  | *pdr5*Δ*pdr10*Δ | 0.87 | (0.013) | 1.74 | n. d. | n. d. | n. d. | 0.096 | n. d. |

**Table S-3** EC_50_ values (μM) of various steroid hormones in yeast strains expressing human sex hormone receptors.

Abbreviation used: n. d., not detectable; n. c., not calculable.

EC_50_ values (μM) shown in parentheses are based on the data listed in Table 1.

**Table S-4** Comparison of responses of yeast strains expressing human sex hormone receptors against known antagonist ligands

| Receptor | Strains | Ligands | | | |
| --- | --- | --- | --- | --- | --- |
|  |  | ICI | | Tam | |
|  |  | IC_50_ (μM) | Maximum inhibition (%) | IC_50_ (μM) | Maximum inhibition (%) |
| ERα | W303a | n. d. | n. a. | 45.6 | 62.0 |
|  | *cwp1*Δ*cwp2*Δ | n. c. | 30.9 | 28.3 | 91.8 |
| ERβ | W303a | n. c. | 17.2 | n. c. | 35.1 |
|  | *cwp1*Δ*cwp2*Δ | 11.4 | 51.1 | 22.3 | 89.8 |
| Receptor | Strains | Ligands | | | |
|  |  | Mif | | Spi | |
|  |  | IC_50_ (μM) | Maximum inhibition (%) | IC_50_ (μM) | Maximum inhibition (%) |
| PR | W303a | n. d. | n. a. | n. d. | n. a. |
|  | *cwp1*Δ*pdr5*Δ | 2.92 | 87.2 | n. c. | 34.0 |
|  | *cwp1*Δ*cwp2*Δ | 21.6 | 84.6 | n. d. | n. a. |
| Receptor | Strains | Ligands | | | |
|  |  | Flu | | Zer | |
|  |  | IC_50_ (μM) | Maximum inhibition (%) | IC_50_ (μM) | Maximum inhibition (%) |
| AR | W303a | 42.8 | 65.2 | 77.8 | 53.3 |
|  | *cwp1*Δ*pdr5*Δ | 18.1 | 82.9 | 73.2 | 53.6 |
|  | *pdr5*Δ*pdr10*Δ | 47.1 | 61.9 | n. c. | 41.1 |

Abbreviation used: IC_50_, 50% effective concentration for inhibition; n. a., not applicable; n. c., not calculable; n. d., not detectable

**Table S-5** Partial agonist activity of known antagonist ligands in yeast strains expressing human sex hormone receptors.

| Receptor | Strains | Ligands | | | |
| --- | --- | --- | --- | --- | --- |
|  |  | ICI | Tam | Ral | MPP |
| ERα | W303a | 1.26 | n. c. | n. c. | n. c. |
|  | *cwp1*Δ*cwp2*Δ | n. d. | 0.10 | 2.51 | 13.4 |
| ERβ | W303a | n. d. | 0.24 | 3.09 | 19.2 |
|  | *cwp1*Δ*cwp2*Δ | n. d. | 0.08 | 0.027 | 2.73 |
| Receptor | Strains | Ligands | | | |
|  |  | Mif | | Spi | |
| PR | W303a | n. d. | | n. d. | |
|  | *cwp1*Δ*pdr5*Δ | n. d. | | 30.8 | |
|  | *cwp1*Δ*cwp2*Δ | n. d. | | n.c. | |
| Receptor | Strains | Ligands | | | |
|  |  | Flu | | Zer | |
| AR | W303a | n. d. | | n. d. | |
|  | *cwp1*Δ*pdr5*Δ | n. c. | | n. c. | |
|  | *pdr5*Δ*pdr10*Δ | 17.9 | | 23.8 | |

Values indicated EC_50_ (μM).

Abbreviation used: n. d. not detectable, n. c. not calculable

**Table S-6** Anti-AR activity of the fourth generation of progestins in yeast.

| Receptor | Strains | Ligands | | | |
| --- | --- | --- | --- | --- | --- |
|  |  | DNG | | DRSP | |
|  |  | IC_50_ (μM) | Maximum inhibition (%) | IC_50_ (μM) | Maximum inhibition (%) |
| AR | W303a | n. c. | 65.3 | n. c. | 58.5 |
|  | *cwp1*Δ*pdr5*Δ | 38.1 | 42.1 | 64.7 | 47.4 |
|  | *pdr5*Δ*pdr10*Δ | 30.2 | 33.4 | n. c. | 59.5 |

Abbreviation used: IC_50_, 50% effective concentration for inhibition (μM); n. c., not calculable
