# Supplementary figures and images for: Construction of reporter gene assays using CWP and PDR mutant yeasts for enhanced detection of various sex steroids
Source: Genes Environ. 2020 May 27;42:20. doi: 10.1186/s41021-020-00159-x (PMC7251871; doi:10.1186/s41021-020-00159-x)

## Slide 1
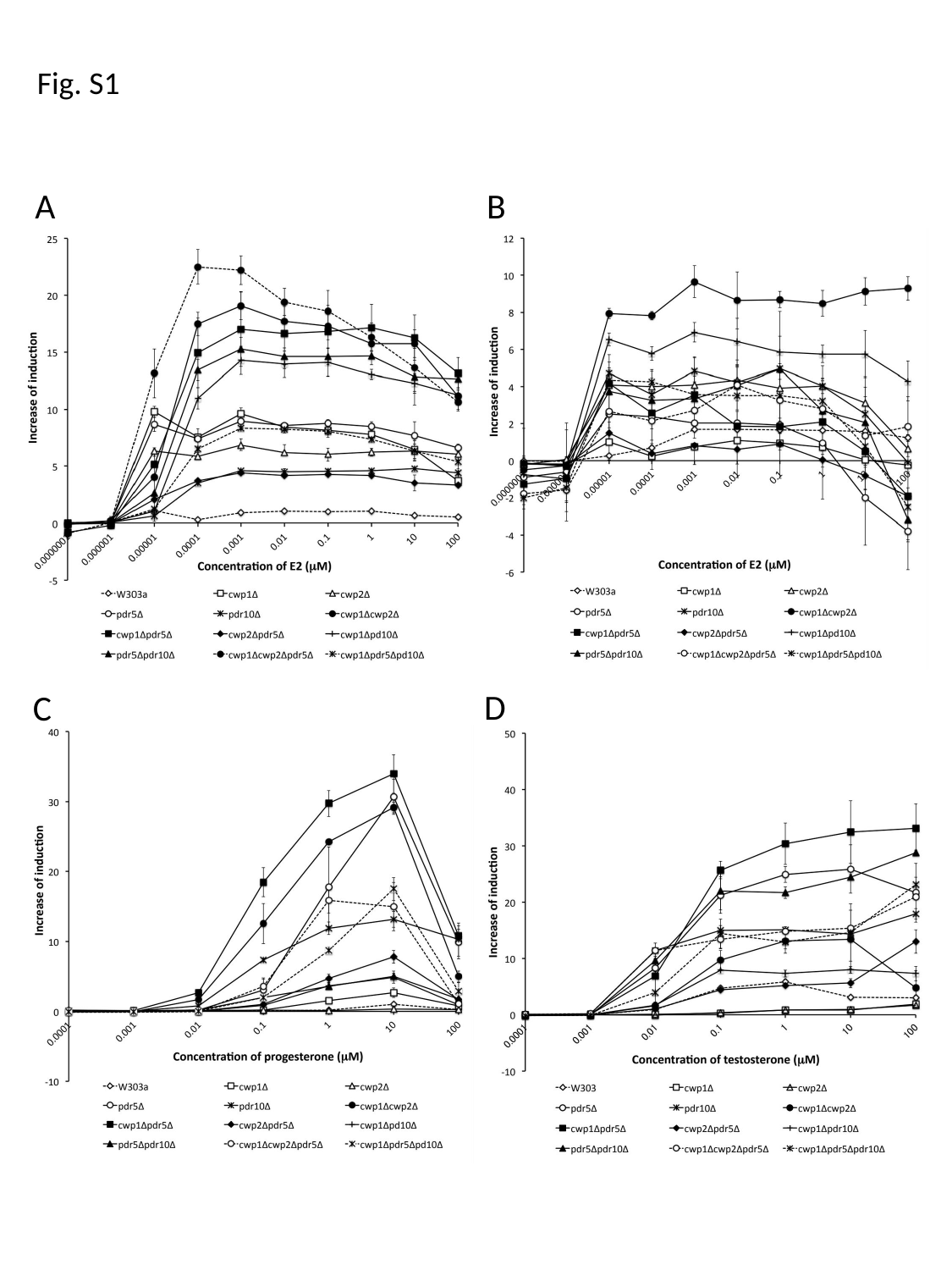

Fig. S1
A
B
D
C

## Slide 2
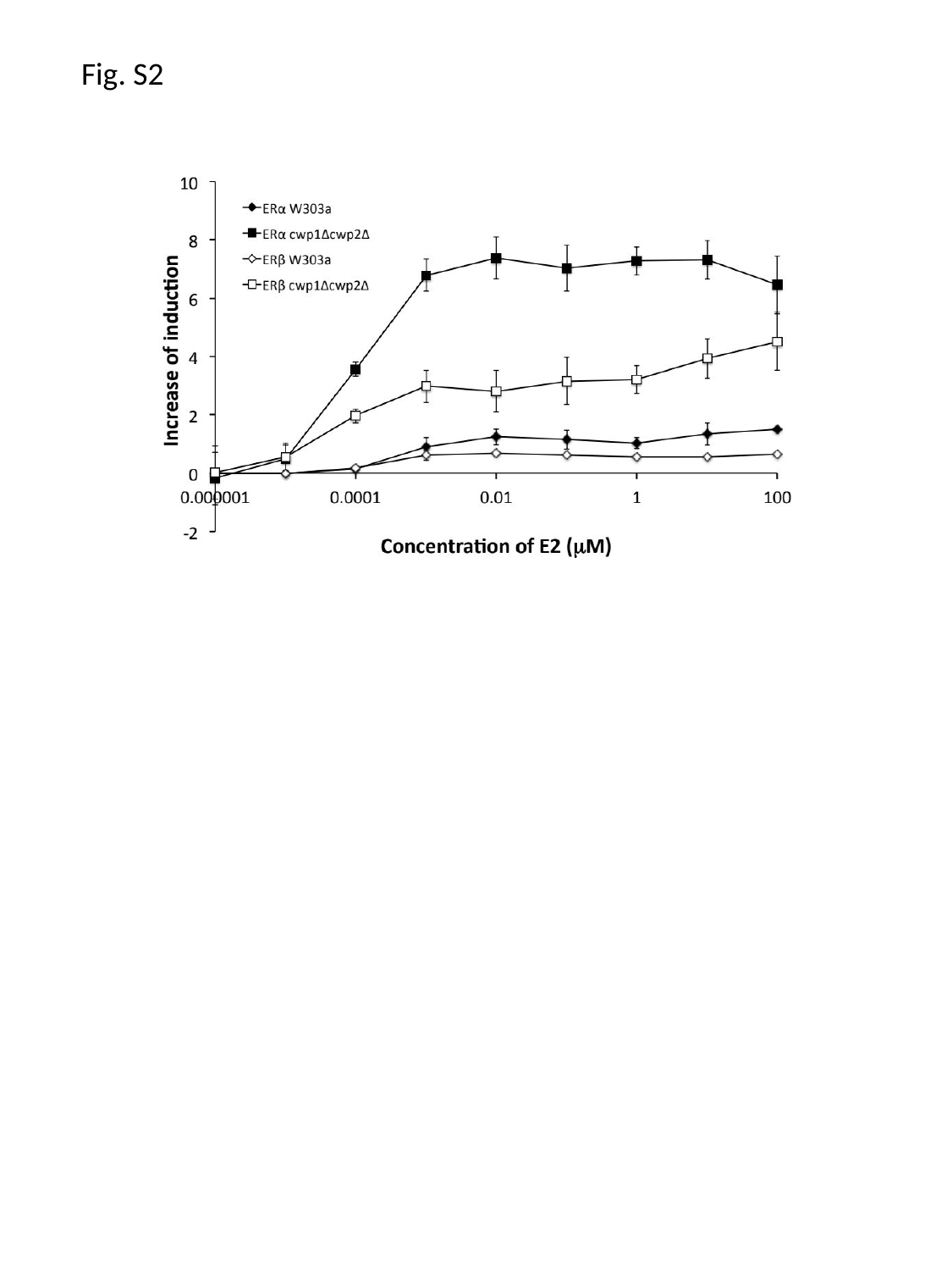

Fig. S2

Supplement: Supplementary file 2 — Additional file 2: Figure S1. Dose-dependent responses to sex steroid hormones in ER, ER, PR, and AR in wild-type W303a and mutants. The reporter assay yeast strains expressing human ER (A), ER (B), PR (C), and AR (D) were established in wild-type yeast W303a and deletion mutants for cell wall mannoproteins (CWP1, CWP2) and/or plasma membrane efflux pumps (PDR5, PDR10). The yeast strains were exposed to E2 (A and B), PS (C), and TS (D), and the ligand-dependent induction of -galactosidase activity was measured. Figure S2. Dose-dependent responses of ER- and ER-expressing yeast strains exposed to E2 in medium containing increased amounts of glucose. ER- and ER-expressing wild-type W303a and cwp1cwp2strains were exposed to E2 in medium containing 1.2% glucose and 0.8% galactose, which was applied for the assay of synthetic estrogens shown in Fig. 3, in order to minimize ligand-independent reporter induction. [file 41021_2020_159_MOESM2_ESM.pptx]
